# The G*α*_o_ Activator Mastoparan-7 Promotes Dendritic Spine Formation in Hippocampal Neurons

**DOI:** 10.1155/2016/4258171

**Published:** 2015-12-31

**Authors:** Valerie T. Ramírez, Eva Ramos-Fernández, Nibaldo C. Inestrosa

**Affiliations:** ^1^Centro de Envejecimiento y Regeneración (CARE), Facultad de Ciencias Biológicas, Pontificia Universidad Católica de Chile, 8331150 Santiago, Chile; ^2^Center for Healthy Brain Ageing, School of Psychiatry, Faculty of Medicine, University of New South Wales, Sydney, Australia; ^3^Centro UC Síndrome de Down, Pontificia Universidad Católica de Chile, Santiago, Chile; ^4^Centro de Excelencia en Biomedicina de Magallanes (CEBIMA), Universidad de Magallanes, Punta Arenas, Chile

## Abstract

Mastoparan-7 (Mas-7), an analogue of the peptide mastoparan, which is derived from wasp venom, is a direct activator of *Pertussis toxin-* (PTX-) sensitive G proteins. Mas-7 produces several biological effects in different cell types; however, little is known about how Mas-7 influences mature hippocampal neurons. We examined the specific role of Mas-7 in the development of dendritic spines, the sites of excitatory synaptic contact that are crucial for synaptic plasticity. We report here that exposure of hippocampal neurons to a low dose of Mas-7 increases dendritic spine density and spine head width in a time-dependent manner. Additionally, Mas-7 enhances postsynaptic density protein-95 (PSD-95) clustering in neurites and activates G*α*_o_ signaling, increasing the intracellular Ca^2+^ concentration. To define the role of signaling intermediates, we measured the levels of phosphorylated protein kinase C (PKC), c-Jun N-terminal kinase (JNK), and calcium-calmodulin dependent protein kinase II*α* (CaMKII*α*) after Mas-7 treatment and determined that CaMKII activation is necessary for the Mas-7-dependent increase in dendritic spine density. Our results demonstrate a critical role for G*α*_o_ subunit signaling in the regulation of synapse formation.

## 1. Introduction 

G proteins are highly expressed in the mammalian brain and play a critical role in the regulation and development of synaptic transmission because they act as transducers for the G protein-coupled receptors (GPCRs) [1]. They are composed of a guanine nucleotide-binding *α* subunit (G*α*) and a *βγ* complex (G*βγ*). In mammals, 20 different G proteins have been described, each composed of one of the 19*α* subunits, one of the 5*β* subunits, and one of the 12*γ* subunits [2]. In the resting state, G*α* is bound to GDP and associated with G*βγ* and a GPCR. This complex is dissociated when G*α* binds to GTP, causing the activation of G*α* and the G*βγ* complex, allowing them to regulate their downstream effectors [3]. G*α* subunits are separated into four families based on sequence homology (G*α*_s_, G*α*_q_, G*α*_i/o_, G*α*_12/13_); each of these proteins activates a different pathway [4].

The majority of excitatory synaptic connections in the central nervous system are located on small dendritic protrusions, which are enriched in signaling molecules and serve to compartmentalize individual postsynaptic structures [5]. In mouse and human brains, the G protein subunits G*α*_o_, G*α*_i_, G*α*_q_, G*α*_z_, G*α*_s_, G*α*_12_, G*α*_13_, and G*α*_14_ are present in postsynaptic densities (PSDs) [6], suggesting a key role of these signal transducer proteins in the synaptic regulation.

To study the specific role of G proteins in the regulation of dendritic spines, we used Mas-7, a potent analogue of the peptide mastoparan, which is obtained from the venom of* Vespula lewisii* [7]. Mas-7 has a substitution of an alanine for a lysine at position 12 [8] and binds to the plasma membrane to form an *α*-helix structure that activates G*α*_o/i_ subunits without requiring the activation of a GPCR [9]. This peptide shows a wide variety of biological effects, including antiviral activity [10], histamine release from mast cells [11], the induction of potent mitochondrial permeability [12], and tumor cell cytotoxicity [13]. However, the effect of Mas-7 in hippocampal neurons has not been studied.

Here, we show that a low dose of Mas-7 activates G*α*_o_, causing the switch from GDP to GTP in hippocampal neurons. Functionally, Mas-7 increases dendritic spine density through a Ca^2+^-dependent mechanism in hippocampal neurons. Mas-7 also activates a variety of Ca^2+^-sensitive proteins, including CaMKII*α*, which is necessary for the increase in dendritic spine density.

These results suggest that G protein activation, especially the G*α*_o_ subunit activation, may contribute to dendritic spine remodeling in neurons.

## 2. Materials and Methods

### 2.1. Reagents

Mas-7 was purchased from Sigma-Aldrich (St. Louis, MO); Fura-2AM from Molecular Probes (Eugene, OR); and KN93 from Calbiochem (San Diego, CA).

### 2.2. Hippocampal Neuronal Culture

Rat hippocampal cultures were prepared from Sprague-Dawley rats of both sexes at embryonic day 18, as previously described [14]. On day two, neurons were treated with 2 *μ*M cytosine arabinoside for 24 h to avoid glial cell growth. Then, the neurons were cultured with Neurobasal medium supplemented with 1% B27 from Invitrogen (Eugene, OR).

### 2.3. Measurements of Intracellular Ca^2+^ in Hippocampal Neurons

Cytosolic Ca^2+^ signals were determined in cells seeded at 160,000 per 35 mm coverslip; the cells were loaded with 4.5 *μ*M Fura-2-AM for 30 min as previously described [15]. The experiments were performed in an isotonic calcium-free solution (in mM): 140 NaCl, 2.5 KCl, 1.7 MgCl_2_, 5 glucose, 0.5 EGTA, and 10 HEPES (305 mOsm/L, pH 7.4 with Tris). An Olympus Spinning Disc IX81 microscope was used in live-imaging experiments recording 1 photo every 5 seconds. The increases in cytosolic Ca^2+^ are represented by the normalized ratio of the fluorescence emitted at 510 nm after excitation at 340 (which determine the probe bound to Ca^2+^) and 380 nm (probe not bound to Ca^2+^) relative to the ratio measured prior to cell stimulation. The integration of the area under the curve was performed with GraphPad Prism5 software (La Jolla, CA) using the first minute before the stimuli application as a baseline.

### 2.4. Immunoprecipitation of Activated G*α*_o_ Subunit

The G*α*_o_ activation assay kit from New East Bioscience (#80901, King of Prussia, PA) was used. The protocol recommended by the manufacturer was employed with modifications. Briefly, neurons at 14 days* in vitro* (DIV) (seeded at 900,000 cells/well) were treated with 1 *μ*M Mas-7 for 5 or 30 min. Then, the cells were lysed with 0.5 mL 1x kit buffer (#30303) and centrifuged at 12,000 ×g 4°C for 10 min. The supernatants were incubated with 1 *μ*L mouse monoclonal antibody specific for G*α*_o_ bound to GTP (active form) (#26907) and 20 *μ*L A/G agarose beads (#30301) for 2 h at 4°C with orbital rotation. As a positive control, untreated neurons were lysed and then incubated with 10 mM GTP*γ*S (#30302) and 10 mM MgCl_2_ for 90 min at RT, and as a negative control, the lysed neurons were incubated with 10 mM GDP (#30304) and 10 mM MgCl_2_. Later, the lysates were washed 3 times and the beads were suspended in 20 *μ*L Laemmli 2x loading buffer and boiled for 5 min. The total level of G*α*_o_ was detected by immunoblotting with a polyclonal anti-G*α*_o_ antibody (#21015, 1 : 1000).

### 2.5. Western Blot

Neurons at 14 DIV were seeded at 400,000 cells/well and treated with 1 *μ*M Mas-7 and were lysed on ice and immediately processed. Immunoblotting was performed as described [16]. The primary antibodies used included mouse anti-CaMKII*α* (sc-5306, 1 : 1000), mouse anti-phospho-Tyr286-CaMKII*α* (sc-32289, 1 : 1000), rabbit anti-PKC*β*II (sc-210, 1 : 1000), rabbit anti-*β*-tubulin (sc-9104, 1 : 1000), mouse anti-GAPDH (sc-32233, 1 : 5000), and rabbit anti-GSK-3*β* (sc-9166, 1 : 1000) from Santa Cruz Biotechnology Inc. (Santa Cruz, CA); rabbit anti-JNK (#9252, 1 : 1000), rabbit anti-phospho-Thr183/Tyr185-JNK (#4668, 1 : 1000), and rabbit anti-phospho-Ser9-GSK-3*β* (#9336, 1 : 1000) from Cell Signaling Technology (Beverly, MA); rabbit anti-phospho-Ser660-PKC*β*II (ab75837, 1 : 10000) and rabbit anti-G*α*_o_ (ab136535, 1 : 5000) from Abcam (Cambridge, MA); mouse anti-PSD-95 (k28/43, 1 : 1000) from UC Davis/NIH NeuroMab Facility; and mouse anti-*β*-actin (11978, 1 : 10000) from Sigma (St. Louis, MO). Equal amounts of protein were loaded (20 *μ*g).

### 2.6. PSD-95 Immunofluorescence and Image Analysis

Neurons at 14 DIV were plated at 35,000 cells/coverslip and treated with 1 *μ*M Mas-7, fixed with a freshly prepared solution of 4% paraformaldehyde plus 4% sucrose in PBS for 20 min at 4°C, permeabilized with 0.2% Triton X-100 for 5 min at room temperature (RT), and then blocked with 1% BSA in PBS (blocking solution) for 30 min at 37°C. This procedure was followed by an overnight incubation at 4°C with anti-PSD-95 (k28/43, 1 : 400) from UC Davis/NIH NeuroMab Facility and Synapsin I (SynI) (sc-20780, 1 : 2000) from Santa Cruz Biotechnology Inc. The neurons were washed with PBS and incubated for 30 min at 37°C with Phalloidin-Alexa-633 and the secondary antibody (Molecular Probes). Images were acquired from 10 microscope fields for each condition with an Olympus Fluoview FV 1000 confocal microscope. To quantify PSD-95 clusters, we used a previously described protocol [17] using NIH ImageJ software (NIH, Baltimore, MD). The synaptic contacts were measured as previously reported [18].

### 2.7. Transfection and Dendritic Spine Morphology Analysis

Hippocampal neurons plated at 60,000 per poly-D-lysine-coated 12 mm glass coverslip were transfected at 10 DIV with an EGFP plasmid (pEGFP-N1, Clontech, Mountain View, CA) using a NeuroMag kit (KC 30800) from OZ Bioscience (Marseille, France) as described previously [19]. First, the neurons were washed for 30 min with Neurobasal medium. Then, 0.8 *μ*g DNA/1.25 *μ*L magnetobeads per cover were mixed and incubated for 15 min in 100 *μ*L Neurobasal medium at RT. Next, the mix was added by drops to the neurons, and the magnetobeads were allowed to enter the cells by use of the magnet for 15 min (37°C, 5% CO_2_). Subsequently, the magnet was removed for 40 min, and, finally, the transfected medium was replaced with fresh medium. At 14 DIV, the neurons were depleted for 2 h with Neurobasal medium without B7 supplement before being treated with 1 *μ*M Mas-7 or 10 *μ*M KN93 plus Mas-7 at different times. An Olympus Fluoview FV 1000 confocal microscope was used to obtain digital confocal stacks from 15 to 20 serial images with a Z step size of 0.25 *μ*m. Dendritic Z-stacks were reconstructed using the super-pass module of Imaris software. Accurate reconstruction of spine head diameter was achieved using the approximate circle algorithm with a threshold of 0.8. Ten neurons were analyzed for each condition. The mean spines length and spines head width of each neurite were reported. Spine density was calculated by measuring the total number of spines per neurite length (spine density/10 *μ*m).

### 2.8. Live-Cell Imaging of Dendritic Spine Morphogenesis

Hippocampal neurons cultured in round 35 mm coverslips at a density of 160,000 cells/coverslip were transfected with EGFP at 11 DIV. Then, at 14 DIV the neurons were placed in the imaging chamber in an isotonic solution (in mM: 1.2 CaCl_2_, 140 NaCl, 2.5 KCl, 0.5 MgCl_2_, 5 glucose, and 10 HEPES (305 mOsm/L, pH 7.4 with Tris)). The EGFP-positive neurons were imaged with an Olympus Spinning Disc IX81 microscope every 5 min for 45 min after the treatment with 1 *μ*M Mas-7. The images were processed and analyzed using ImageJ software.

### 2.9. Statistical Analysis

Statistical analysis was performed using Prism 5 software. The values are expressed as the mean ± standard error of the mean. The statistical significance of differences was assessed with one-way ANOVA with Bonferroni's posttest for multiple comparisons and with Student's *t*-test for comparisons between two conditions (*p* < 0.05 was considered significant). The number of independent experiments is indicated in the corresponding figure legends.

## 3. Results

### 3.1. Mas-7 Activates the G*α*_o_ Subunit in Hippocampal Neurons

To determine whether the Mas-7 peptide activates G proteins, specifically the G*α*_o_ subunit in cultured rat hippocampal neurons, we treated them with Mas-7 and then performed immunoprecipitation assays, using a commercial specific antibody that recognizes G*α*_o_ bound to GTP (G*α*_o_-GTP), the active form of the G*α*_o_ protein, in combination with SDS-PAGE and immunoblotting. Additionally, we immunoprecipitated G*α*_o_-GTP from lysates of hippocampal neurons that were incubated with nonhydrolyzable GTP (GTP*γ*S) or with GDP as a positive and negative control, respectively.

**Figure 1:**
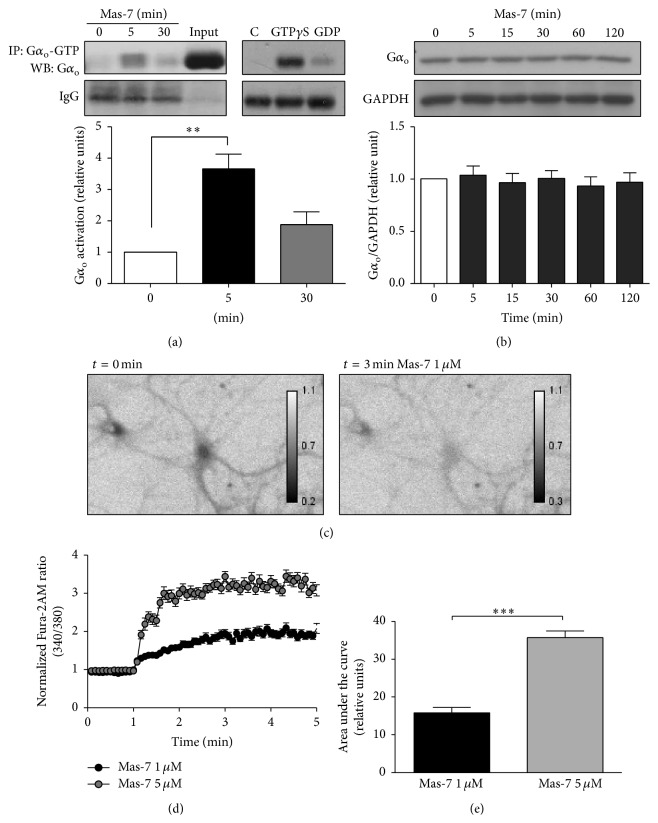
Mas-7 activates the G*α*_o_ subunit and increases the intracellular Ca^2+^ concentration in hippocampal neurons. (a)* Left panel*, 14 DIV hippocampal neurons were stimulated with 1 *μ*M Mas-7 for 0, 5, or 30 min. The neurons were lysed and incubated with anti-G*α*_o_-GTP for 2 h and then analyzed by immunoblotting using an anti-G*α*_o_ antibody (*n* = 4). The input lane corresponds to a lysate sample before the immunoprecipitation. ^*∗∗*^ *p* < 0.01.* Right panel,* lysates from untreated (control) hippocampal neurons were incubated with GTP*γ*S as a positive control or with GDP as a negative control for 90 min at RT. Then, the G*α*_o_-GTP was immunoprecipitated and analyzed by western blotting to determine the total level of G*α*_o_. The IgG band shows that an equal amount of antibody was used for the immunoprecipitation. (b) Representative western blot and quantification of the total level of G*α*_o_ in 14 DIV neurons incubated for different periods of time with 1 *μ*M Mas-7. GAPDH was used as a loading control (*n* = 4). (c) Ratio images (340/380) of the Fura-2AM probe from hippocampal neurons under basal conditions (*t* = 0) or after 3 min of 1 *μ*M Mas-7 treatment. (d) Quantification of measurements of the intracellular Ca^2+^ increase in hippocampal neurons bathed in a Ca^2+^-free solution with different concentrations of Mas-7 (*n* = 3, 70–79 neurons, each condition). (e) Area under the curve of the Ca^2+^ increase after Mas-7 treatment. ^*∗∗∗*^ *p* < 0.001.

Our data show that Mas-7 induced activation of the G*α*_o_ subunit in cultured hippocampal neurons after 5 min of exposure (Figure 1(a),* left panel*); the activation then declined at 30 min. By contrast, Mas-7 was unable to increase the total G*α*_o_ protein level, even after 2 h of treatment (Figure 1(b)). Furthermore, incubation with GTP*γ*S produced an increase in the activation of G*α*_o_, and GDP incubation decreased the activation (Figure 1(a),* right panel*). These findings show that Mas-7 produces rapid activation of G*α*_o_ subunit, suggesting that the G*α*_o_-dependent signaling cascade is also activated.

### 3.2. Mas-7 Increases the Intracellular Calcium Concentration in Hippocampal Neurons

In different cell types, mastoparan and Mas-7 produce an increase in intracellular calcium (Ca^2+^) [20, 21]. For example, in rat cerebellar granule neurons, 15 *μ*M mastoparan produces a robust elevation in intracellular Ca^2+^ [22]. To assess whether Mas-7 generates a similar effect in cultured hippocampal neurons, we treated them with Mas-7 at a lower dose (1–5 *μ*M) to avoid a toxic effect. To quantify the response of individual neurons to Mas-7, we used Fura-2 AM in a Ca^2+^-free solution and measured the fluorescence emitted by the probe at 510 nm after the 340/380 excitation in a live imaging experiments. In Figure 1(c) (Fura-2 AM 340/380 ratio images), it is observed that Mas-7 increased the Ca^2+^ concentration in the soma and dendrites of the hippocampal neurons. Additionally, Mas-7 induced a large elevation of Ca^2+^ in a concentration-dependent manner (Figure 1(d)). There was a significant difference between the Ca^2+^ elevations induced by 1 and 5 *μ*M (Figure 1(e)). This finding suggests that the activation of G*α*_o_ by Mas-7 produces the release of Ca^2+^ from internal cellular stores in hippocampal neurons.

### 3.3. Mas-7 Activates CamKII, PKC, and JNK in Mature Hippocampal Neurons

Because Mas-7 increased the Ca^2+^ concentration in hippocampal neurons, we evaluated whether Mas-7 could activate Ca^2+^-dependent kinases, such as CaMKII*α* and PKC*β*II. Previous reports in neuronal and nonneuronal cells have shown that mastoparan can induce a rise in Ca^2+^ via a phospholipase C- (PLC-) dependent mechanism [7, 22]. The ability of Mas-7 to activate these kinases was determined using anti-phospho-Tyr286-CaMKII*α* or anti-phospho-Ser-660-PKC*β*II antibodies in mature hippocampal neurons.

**Figure 2:**
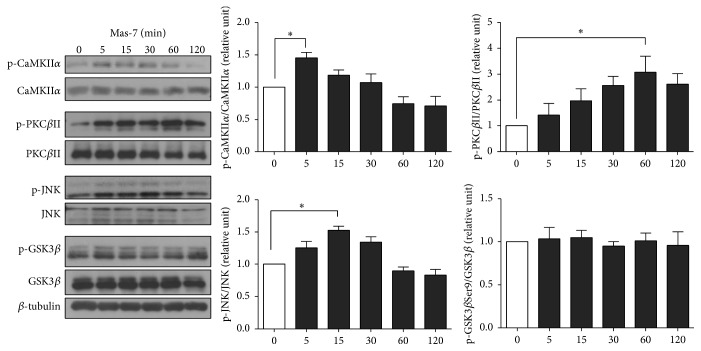
Mas-7 activates CaMKII, PKC, and JNK in hippocampal neurons. Representative western blot and quantification of total and phosphorylated levels of CaMKII*α* (*n* = 4), PKC*β*II (*n* = 4), JNK (*n* = 3), and GSK-3*β* (*n* = 3) in 14 DIV hippocampal neurons incubated with 1 *μ*M Mas-7 for different times. *β*-tubulin was used as a loading control. ^*∗*^ *p* < 0.05.

As illustrated in Figure 2, Mas-7 stimulated the activity of CaMKII*α* in a time-dependent manner. We found that hippocampal neurons treated with Mas-7 showed a slight but significant increase in the active form of CaMKII*α* after 5 min. Then, the phosphorylation of CaMKII*α* decreased and remained at a similar level after 1-2 h with respect to the control condition. In addition, Mas-7 treatment increased PKC phosphorylation after 5 min, with a peak at 60 min (Figure 2). The total levels of PKC were not affected by Mas-7 treatment. Moreover, we analyzed the effect of Mas-7 on the phosphorylation state of other kinases, such as JNK and glycogen synthase kinase-3*β* (GSK-3*β*). Mas-7 also induced an increase in JNK phosphorylation (p-JNK-Thr183/Tyr185) after 15 min, mainly of the JNK1 isoform, which corresponds to the lower band. However, hippocampal neurons exposed to Mas-7 did not show any change in the phosphorylation of GSK-3*β* at serine 9 (p-GSK-3*β*-Ser9), even after 2 h of treatment. These findings indicate that G*α*_o_ activation by Mas-7 promotes the activation of CaMKII, PKC, and JNK in hippocampal neurons.

### 3.4. New PSD-95 Clusters Are Induced by Mas-7 Treatment in Hippocampal Neurons

PSD-95 is a scaffold protein that plays a key role in synapse organization, during dendritic spine formation [23]. Here, we examined whether the Mas-7 peptide could regulate the postsynaptic region in mature hippocampal neurons. Specifically, we analyzed whether Mas-7 would induce an increase in PSD-95 clustering in 14 DIV neurons by measuring PSD-95 density and the area of the clusters by immunofluorescence, as described previously [17].

**Figure 3:**
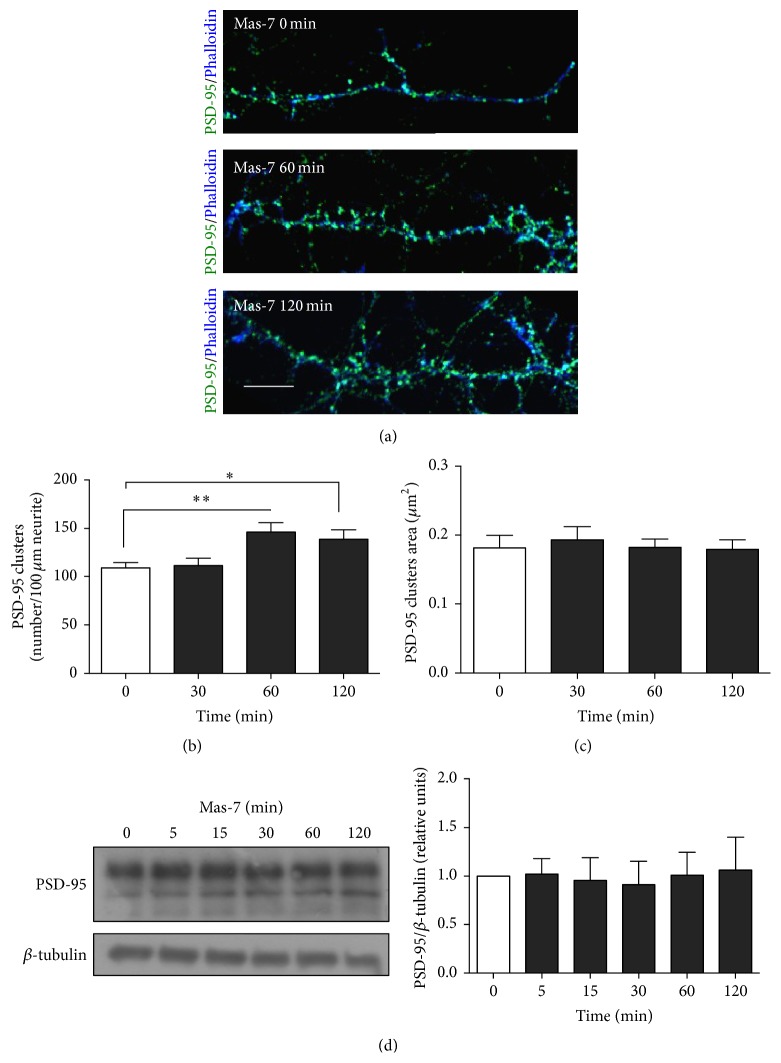
Mas-7 increases the number of PSD-95 clusters in hippocampal neurons. (a) Representative immunofluorescence images for PSD-95 (green) and Phalloidin (blue) in hippocampal neurons exposed to Mas-7 for 0, 60, and 120 min. Scale bar = 6 *μ*m. (b) Quantification of number of PSD-95 clusters/100 *μ*m of neurite (*n* = 4). (c) Quantification of PSD-95 cluster area (*n* = 4). (d) Western blot and quantification of total levels of PSD-95 in hippocampal neurons exposed to Mas-7 for different lengths of time (*n* = 3). ^*∗∗*^ *p* < 0.01 and ^*∗*^ *p* < 0.05.

The effect of Mas-7 in the clustering of PSD-95 was evaluated, and a time-dependent increase in the number of PSD-95 clusters was observed. As indicated in Figures 3(a) and 3(b), PSD-95 clustering increased approximately 50% after 60 min of Mas-7 treatment and remained elevated at 2 h in comparison with the control. To evaluate whether the increase in PSD-95 density was due to an increase in the expression of PSD-95, we measured the area of PSD-95 clusters and the levels of PSD-95 protein in total extracts. Mas-7 did not change the area of the PSD-95 clusters (Figure 3(c)) or the PSD-95 total protein level (Figure 3(d)) over the same temporal course for which we observed the increase in the number of PSD-95 clusters, suggesting redistribution of existing PSD-95 proteins, rather than changes in expression.

Thus, our findings suggest that G*α*_o_ activation by Mas-7 triggers PSD-95 remodeling that promotes clustering and additional postsynaptic assembly, without changing the expression of the PSD-95 protein.

### 3.5. Mas-7 Changes the Morphology and Density of Dendritic Spines in Hippocampal Neurons

**Figure 4:**
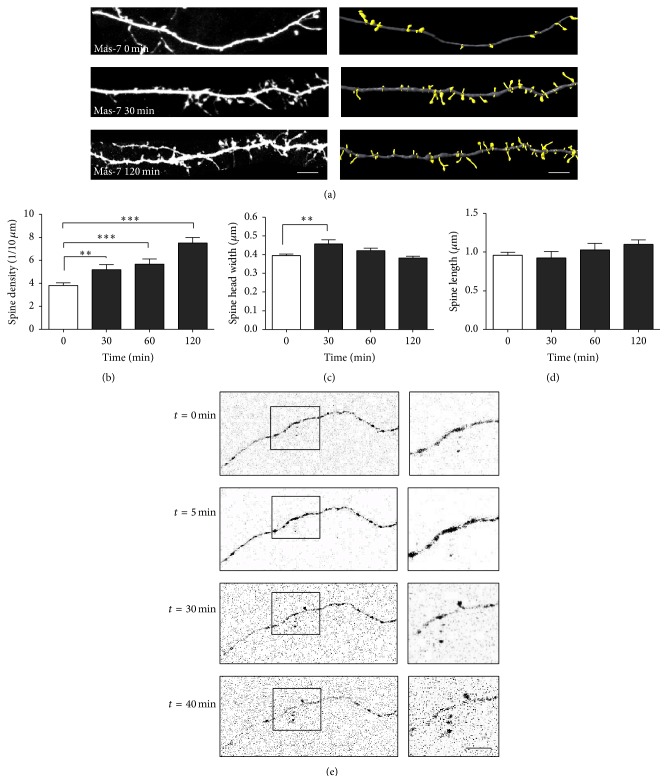
G*α*_o_ activation by Mas-7 regulates dendritic spine morphogenesis. (a)* Left panel*, representative images of 14 DIV hippocampal neurons treated with Mas-7 for 0, 30, or 120 min.* Right panel*, 3D reconstructions of neurites. Scale bar = 5 *μ*m. (b) Quantification of dendritic spine density. (c) Quantification of spine head width. (d) Quantification of spine length (*n* = 3). ^*∗∗∗*^ *p* < 0.001, ^*∗∗*^ *p* < 0.01, and ^*∗*^ *p* < 0.05. (e) Mas-7 induces* de novo* formation of dendritic protrusions. Live cell time-lapse imaging of the formation of dendritic spines in response to Mas-7 in 14 DIV hippocampal neurons. A dendrite of an EGFP-transfected neuron is shown before and after 5, 30, and 40 min of treatment with Mas-7. Scale bar = 2 *μ*m.

The presence of PSD-95 clusters in excitatory neurons is well correlated with the number of mature dendritic spines [24]. For this reason, we attempted to determine the role of Mas-7 in dendritic spine formation by transfecting mature hippocampal neurons with EGFP at 10 DIV and then exposed them to 1 *μ*M Mas-7 at different times at 14 DIV. Dendritic spine protrusions below 3 *μ*m in length were analyzed using Imaris software to measure spine length, width, and density. Hippocampal neurons exposed to Mas-7 exhibited increased dendritic spine density after 30 min and 1 h (Figure 4(a)), with a peak at 2 h. Additionally, Mas-7 increased spine head width after 30 min (Figure 4(c)); however, the length of the spines was not significantly affected (Figure 4(d)). An increase in dendritic spine head width has been related to the strength of synaptic transmission [25], which suggests that Mas-7 might also regulate that process.

To provide additional evidence of the effect of Mas-7 in the development of dendritic spine protrusions, live cell time-lapse imaging of the formation of dendritic spines was performed. EGFP-transfected neurons were treated for 45 min with Mas-7 (Figure 4(e)), and we determined that Mas-7 produced* de novo* formation of a dendritic spine. This new protrusion presented a recognizable head and appeared after 30 min of treatment.

Together, these results suggest that the activation of G*α*_o_ has a regulatory effect on spine morphogenesis in hippocampal neurons.

### 3.6. The Activation of CaMKII*α* Is Involved in the Dendritic Spine Density Increase Induced by Mas-7

Mas-7 is capable of activating CaMKII*α* very rapidly; thus, we sought to establish whether this activation was required to produce the increase in dendritic spines density to understand the cellular mechanism of Mas-7. Interestingly, CaMKII*α* has been related to dendritic spine formation and regulation [26].

**Figure 5:**
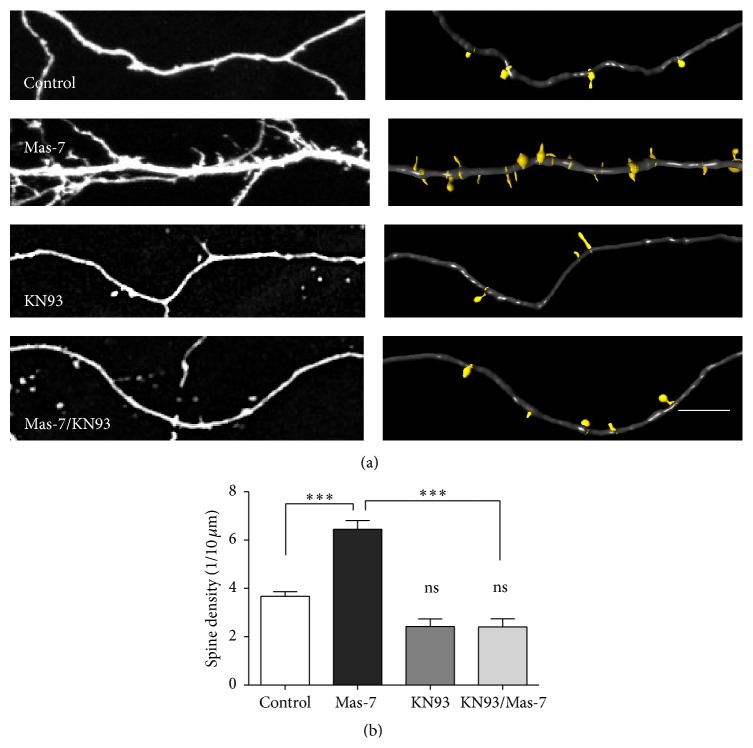
Mas-7 induced spinogenesis through the activation of CaMKII. (a)* Left panel*, representative images of 14 DIV hippocampal neurons untreated (control) or treated with or without Mas-7 for 2 h and coincubated with KN93.* Right panel*, representative 3D reconstructions. (b) Quantification of dendritic spine density for all of the conditions (*n* = 4). Scale bar = 5 *μ*m. ^*∗∗∗*^ *p* < 0.001. ns = no significant difference.

We used KN93, a classic inhibitor of CaMKII activation [27]. In hippocampal neurons, KN93 completely blocked the increase of dendritic spine density induced by Mas-7 at 2 h, without affecting density when applied alone (Figures 5(a) and 5(b)). These results indicate that the activation of CAMKII*α* by the increase in intracellular Ca^2+^ concentration is required for the regulation of spinogenesis induced by Mas-7.

### 3.7. Mas-7 Induces Synaptic Contacts in Hippocampal Neurons

**Figure 6:**
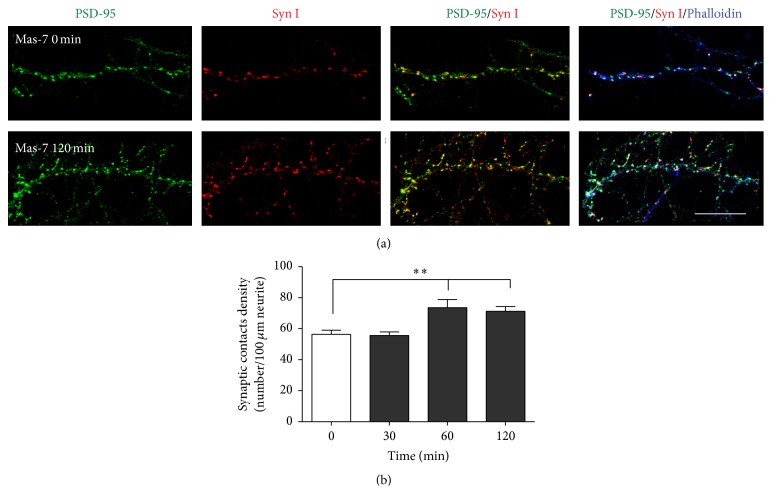
Mas-7 induces the formation of synaptic contacts. (a) Representative images of hippocampal neurons at 14 DIV treated with 1 *μ*M Mas-7 for 0 or 120 min. PSD-95 (green), SynI (red), and Phalloidin (blue) immunofluorescence. Scale bar = 4 *μ*m. (b) Quantification of the density of synaptic contacts (10 neurons were analyzed in each experiment). ^*∗∗*^ *p* < 0.01.

Our previous findings of Mas-7 regulation of spine formation as well as of PSD-95 cluster remodelling (Figures 3 and 4) led us to suggest that Mas-7 is a regulator of the synapse. To address this alternative, hippocampal neurons at 14 DIV were incubated for 1 or 2 h with Mas-7. Treatment with 1 *μ*M Mas-7 significantly increased the number of synaptic contacts after 1-2 h of treatment (Figure 6). The synaptic contacts were observed by the staining for PSD-95 (green) and for the presynaptic protein Syn I (red), where both stains are facing directly. Interestingly, our results suggest that Mas-7 rapidly increases the number of PSD-95 puncta at 1 and 2 h and simultaneously increases the number of synaptic contacts.

## 4. Discussion

In the present work, we studied the effect of Mas-7, a peptide used to pharmacologically activate G proteins, on synaptic structure.

First, our findings show that the G*α*_o_ subunit is activated in hippocampal neurons by Mas-7 treatment, which is consistent with previous data obtained in other cellular contexts, through a mechanism similar to the action of GPCRs [9, 28]. The rapid activation of G*α*_o_ occurred after 5 min of Mas-7 exposure in hippocampal neurons, as its analogue mastoparan, because it promotes the dissociation of GDP and enhances the GTP binding [29]. Additionally, mastoparan increases the intrinsic GTPase activity of G proteins, which suggests that the active state of G*α*_o_ is transient [30]. We observed that, after 30 min of treatment, the activation of G*α*_o_ decayed, which is consistent with an increase in GTPase activity.

Furthermore, it is possible that Mas-7 can activate other G proteins subunits in hippocampal neurons, such as G*α*_i_, because mastoparan has been shown to be able to activate both these subunits in a biochemical assay [9].

In hippocampal neurons, Mas-7 presents an immediate effect, increasing the intracellular Ca^2+^ in a concentration-dependent manner, as previously reported in neuroblastoma cells [21] and in neutrophils [20]. This increase remained at least 4-5 min after treatment with Mas-7. It is known that, in addition to being an activator of G proteins, Mas-7 is an inhibitor of ATPase activity from the endoplasmic reticulum [31], which could explain the sustained rise. This elevation in the levels of Ca^2+^ led to the activation of CaMKII*α* and PKC*β*II, two Ca^2+^-dependent kinases, as well as JNK. The activation of CaMKII was fast, but Mas-7 activated PKC after 1 h. It would be interesting to study how they regulate the biological effects of Mas-7.

On the other hand, it has been suggested that mastoparan treatment can produce apoptosis in cerebellar granule cells through Ca^2+^ release, probably via activation of a transduction pathway involving PLC and IP_3_, using 10–20 *μ*M concentration [22]. In the present study, Mas-7 mimicked the increase in Ca^2+^ from internal stores; however, we used a lower concentration of Mas-7 for a shorter period of time, which did not replicate the apoptotic effect in hippocampal neurons. Higher concentrations of Mas-7 can probably also trigger a similar apoptotic effect in mature hippocampal neurons.

Previous studies have established that G*α*_o_ is highly expressed in the central and peripheral nervous systems, where it represents approximately 1% of membrane proteins [32]. Additionally, the G*α*_o_ subunit has been linked to cognitive and memory functions in the adult brain. The corresponding knockout mice exhibit neurological impairments, such as reduced motor control, hyperactivity, hyperalgesia, and a shortened lifespan [33]. Moreover, G*α*_o_ is required for the formation of associative memory in mushroom body neurons in* D. melanogaster* [34].

Functionally, we observed that Mas-7 was able to modulate the postsynaptic region in the mammalian CNS. In the postsynaptic region, the regulation of dendritic spines is a key process in neuronal plasticity and memory. Dendritic spines undergo structural modifications in response to a diverse range of stimuli [35]. Here, we showed that Mas-7 can regulate dendritic spine formation, increase dendritic spine density and head width, and increase PSD-95 clustering. Although there are several studies that support a model in which PSD-95 is recruited in an activity-dependent manner to new spines, where it contributes to the stabilization of nascent spines [36, 37], we sought to study whether these new spines could form synapses. We found that Mas-7 also increased the number of synaptic contacts, which suggests that G*α*_o_ activation is able to generate functional synapses. Further studies are required to demonstrate whether the increase in the dendritic spine density induced by Mas-7 has a positive impact on memory and learning* in vivo*.

Effects of Mas-7 treatment on other aspects of neuronal development have been previously demonstrated, such as a significant increase in axonal growth in hippocampal neurons [38], which suggests that activation of the G*α*_o_ subunit generates several structural effects that could produce cytoskeleton remodeling. In particular, we propose that the Ca^2+^ increase produced by Mas-7 and the subsequent activation of the downstream Ca^2+^-sensitive kinases PKC*β*II and CaMKII*α* as well as JNK can explain the dendritic spine remodeling. These kinases are known regulators of dendritic spine morphology [26, 39, 40], but, particularly, it is known that CaMKII*α* is highly expressed in spines and is important for long-term potentiation (LTP) [41]. We found that blocking the activity of CaMKII*α* prevents the Mas-7-dependent increase in dendritic spine density, helping to elucidate the mechanism by which Mas-7 acts. Certainly, understanding the role of PKC and JNK could provide us with a global vision of the involvement of G*α*_o_ signaling in spine remodeling and synapse formation.

All of these findings indicate that G*α*_o_ might be important for the regulation and maintenance of synapses. We suggest a mechanism, in which the activation of this G protein subunit increases the levels of Ca^2+^, activates CaMKII*α* to remodel the postsynaptic region, and ultimately leads to the formation of synaptic contacts.

## 5. Conclusion

In this work, we demonstrated that the peptide Mas-7 produced several biological effects in mature hippocampal neurons, including activation of G*α*_o_ signaling and of CaMKII*α*, JNK, and PKC*β*II. Functionally, our results suggest that Mas-7 causes dendritic spine remodeling, increases the number of spines, and recruits PSD-95 protein into spines to produce functional synapses.
